# Clinical Factors and Outcomes When Real-World Heart Teams Overruled STS Risk Scores in TAVR Cases

**DOI:** 10.1155/2022/9926423

**Published:** 2022-06-25

**Authors:** Jackson M. King, Morgan T. Black, Ruyun Jin, Gary L. Grunkemeier, Branden R. Reynolds, Brydan D. Curtis, Robert W. Hodson, Erika A. Strehl, Sameer A. Gafoor, Matthew D. Forrester, Emily J. Cox, Michael E. Ring

**Affiliations:** ^1^Elson S. Floyd College of Medicine, Washington State University, Spokane, WA, USA; ^2^Center for Cardiovascular Analytics, Research and Data Science, Providence Heart Institute, Providence St. Joseph Health, Portland, OR, USA; ^3^Providence Sacred Heart Medical Center, Spokane, WA, USA; ^4^Providence St. Vincent Medical Center, Portland, OR, USA; ^5^Heart Institute, Providence St. Joseph Health, Renton, WA, USA; ^6^Swedish Heart and Vascular Institute, Swedish Medical Center, Seattle, WA, USA; ^7^Cardiovascular Center Frankfurt, Frankfurt, Germany; ^8^Providence Medical Research Center, Spokane, WA, USA

## Abstract

**Objectives:**

This study was conducted to determine why heart teams recommended transcatheter aortic valve replacement (TAVR) versus surgical AVR (SAVR) for patients at low predicted risk of mortality (PROM) and describe outcomes of these cases.

**Background:**

Historically, referral to TAVR was based predominately on the Society of Thoracic Surgeons (STS) risk model's PROM >3%. In selected cases, heart teams had latitude to overrule these scores. The clinical reasons and outcomes for these cases are unclear.

**Methods:**

Retrospective data were gathered for all TAVR and SAVR cases conducted by 9 hospitals between 2013 and 2017.

**Results:**

Cases included TAVR patients with STS PROM >3% (*n* = 2,711) and ≤3% (*n* = 415) and SAVR with STS PROM ≤3% (*n* = 1,438). Leading reasons for recommending TAVR in the PROM ≤3% group were frailty (57%), hostile chest (22%), severe lung disease (16%), and morbid obesity (13%), and 44% of cases had multiple reasons. Most postoperative and 30-day outcomes were similar between TAVR groups, but the STS PROM ≤3% group had a one-day shorter length of stay (2.5 ± 3.4 vs. 3.5 ± 4.7 days; *p* ≤ 0.001) and higher one-year survival (91.6% vs. 86.0%, *p*=0.002). In patients with STS PROM ≤3%, 30-day mortality was higher for TAVR versus SAVR (2.0% vs. 0.6%; *p* < 0.001).

**Conclusions:**

Heart teams recommended TAVR in patients with STS PROM ≤3% primarily due to frailty, hostile chest, severe lung disease, and/or morbid obesity. Similar postoperative outcomes between these patients and those with STS PROM >3% suggest that decisions to overrule STS PROM ≤3% were merited and may have reduced SAVR 30-day mortality rate.

## 1. Introduction

Over the past decade, surgical aortic valve replacement (SAVR) has largely been supplanted by transcatheter aortic valve replacement (TAVR) as the preferred intervention for most patients with severe symptomatic aortic stenosis (AS) [1,2]. Eligibility for TAVR in the United States is governed by federal regulations, which have changed twice in the last decade. Prior to 2016, federal regulations reserved TAVR for patients at high or prohibitive risk for SAVR predominately based on the Society of Thoracic Surgeons-predicted risk of mortality (STS PROM) model for SAVR. In 2016, after clinical trials showed that TAVR was either superior or noninferior to SAVR in the treatment of patients with severe symptomatic AS at intermediate risk of postoperative mortality [3,4], TAVR eligibility was expanded to include intermediate-risk patients (STS PROM scores >3%). Following comparable randomized studies in patients at low risk for SAVR (STS PROM < 3–4%), eligibility changed again in 2019 to permit TAVR in patients with STS PROM scores ≤3% [5–7].

STS PROM scoring algorithms do not incorporate all the clinical factors that clinicians evaluate when determining patient risk, such as the presence of porcelain aorta. Thus, federal guidelines have always afforded clinical discretion to local heart teams such that they may recommend TAVR if it is believed that a patient's individualized risk is not adequately represented by the STS PROM risk model. However, little is known about the effects of this discretionary decision-making on the postoperative outcomes of TAVR in real-life populations. In particular, reasons for recommending TAVR in patients with STS PROM scores ≤3% have not been reported, and there are few reports of postoperative outcomes in such cases. The purpose of this study was to identify the specific clinical factors that led local heart teams to overrule STS PROM scores ≤3% and to summarize clinical outcomes for these cases. In addition, we sought to compare the baseline characteristics and in-hospital outcomes of AS patients with STS PROM ≤3% whom the local heart team referred for SAVR, anticipating exclusion of patients with higher risk clinical conditions not incorporated by the STS PROM risk model might lead to improved SAVR outcomes.

## 2. Materials and Methods

### 2.1. Ethics Statement

This retrospective data collection study was approved by the Providence Health Care Institutional Review Board. The Institutional Review Board waived the requirement for informed consent due to the retrospective nature of the study.

### 2.2. Data Collection

Patients who underwent nonclinical trial TAVR and SAVR between 2013 and 2017 were identified from 9 hospitals in the Providence St. Joseph Health System across 5 western states (Alaska, California, Montana, Oregon, and Washington). In those hospitals that were not performing TAVR at the beginning of the time period, inclusion of SAVR patients did not start until the date of the first TAVR at that hospital. Only SAVR patients with STS PROM ≤3% were included in this study.

Demographic and clinical data for TAVR patients, including STS PROM scores, were gathered from the STS/American College of Cardiology Transcatheter Valve Therapy (TVT) Registry™ [8]. The outcomes including significant cardiac event, stroke, acute kidney injury (stage 3), bleeding (disabling or life threatening), vascular access site complications, and device complications were defined by TVT Registry™ V2.1 Institutional Outcomes Report Companion Guide for TAVR Procedures. Similar data for SAVR patients was obtained from the STS Adult Cardiac Surgery Database [9]. The outcomes followed TVT definitions, except that the variables significant cardiac events, bleeding (disabling or life threatening), vascular complications, and device complications were not available. One-year survival status for TAVR cases was determined by using data from the TVT Registry™, electronic medical records, and the Social Security Death Index. Accurate survival data for the SAVR group were only possible to 30 days as per the registry collection requirements.

Based on the type of procedure and the 2008 STS PROM risk model, which was the model in use during the data collection period, patients were classified into one of the three subgroups: TAVR with STS PROM ≤3%, TAVR with STS PROM >3%, and SAVR with STS PROM ≤3%.

Missing data were handled as described in Supplementary Materials 1.

### 2.3. Surveys to Heart Teams

Local heart teams at each hospital agreed to complete surveys for all cases in which TAVR was recommended for patients with STS PROM ≤3% (Supplementary Materials 2; survey instrument). Study surveys were completed by TAVR coordinators or a designee at each site. Surveys were collected by encrypted e-mail, and answers were collated in Microsoft Excel®. Surveys prompted respondents to list the specific clinical factor(s) driving the local heart team's recommendation, and respondents were allowed to select more than one reason per case. Unclear answers were resolved by querying the electronic medical record.

### 2.4. Statistical Analysis

Continuous data were summarized as mean ± standard deviation (SD) or median (interquartile range (IQR)) and compared between groups by the *t*-test or Wilcoxon rank sum test, as appropriate. Categorical data were presented as proportions and compared by the chi-squared test or Fisher's exact test, as appropriate. Long-term survival was analysed with the Kaplan–Meier estimator and compared using the log-rank test. Long-term readmission with competing risk to mortality was represented by the cumulative incidence function, with pointwise confidence intervals (CIs) obtained using the method proposed by Choudhury, and compared by Gray's test [10,11]. Statistical analyses were performed by using *R* version 3.5.1 [12]. The *R* packages *survival* and *cmprsk* were used. Significance was set at *p* < 0.05.

## 3. Results

### 3.1. Patients

**Figure 1:**
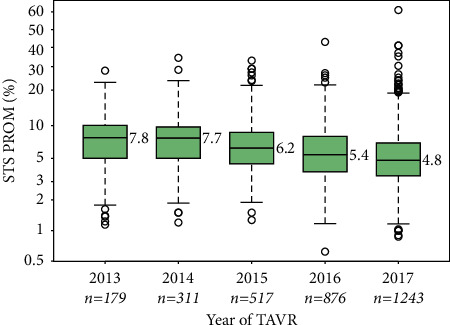
STS PROM of patients undergoing transcatheter aortic valve replacement procedures. Society of Thoracic Surgeons predicted risk of mortality (STS PROM) scores for all transcatheter aortic valve replacement (TAVR) procedures performed in nine hospitals in a five-year period are shown. Values are displayed as medians (middle bars) and interquartile ranges (solid boxes) on a logit scale.

**Figure 2:**
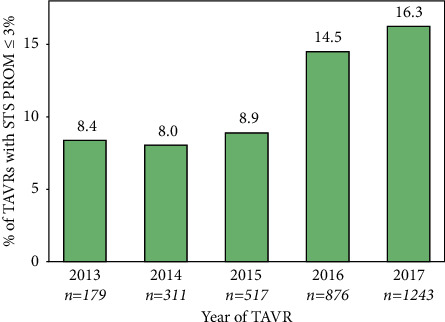
Percentage of transcatheter aortic valve replacement procedures performed in patients with STS PROM ≤3%. Percentage of overall transcatheter aortic valve replacement (TAVR) procedures that were performed in patients with STS PROM ≤3% by year of procedure is shown.

A total of 3,126 patients underwent nonclinical trial TAVR in the Providence St. Joseph Health System from 2013 through 2017. Of these, 415 (13.3%) were identified by the local heart team as having a calculated STS PROM ≤3%. As median STS PROM scores decreased over the data collection period from 7.8 in 2013 to 4.8 in 2017, the number of TAVR cases increased markedly, from 179 in 2013 to 1,243 in 2017 (Figure 1). Similarly, the incidence of patients with an STS PROM ≤3% increased from 8.4% in 2013 to 16.3% in 2017 (Figure 2).

The median risk scores were 6.0 for the STS PROM >3% TAVR group and 2.3 for the STS PROM ≤3% group (Table 1). Compared to patients with STS PROM >3%, TAVR patients with STS PROM ≤3% were significantly younger (74.4 ± 8.6 vs. 82.2 ± 8.4 years, respectively, p < 0.001) and included more males (69.6% vs. 51.1%, respectively, p < 0.001). The STS PROM ≤3% TAVR group had a significantly better ejection fraction, higher mean gradients across the aortic valve, and a lower incidence of moderate to severe mitral regurgitation. Compared to the STS PROM >3% TAVR group, there was a higher proportion of bicuspid aortic valve morphology in the STS PROM ≤3% group (6.8% vs. 2.4%, *p* < 0.001).

During the same time period, a total of 1,438 patients with STS PROM ≤3% underwent isolated SAVR at these same facilities. Compared to STS PROM ≤3% TAVR patients, SAVR patients had a lower median STS PROM (1.3 vs. 2.3; *p* < 0.001) and were also younger (64.4 ± 11.8 vs. 74.4 ± 8.6 years, *p* < 0.001). Many comorbidities such as diabetes, prior stroke, peripheral arterial disease, atrial fibrillation/flutter, prior myocardial infarction, and advanced chronic lung disease were more prevalent in the STS PROM ≤3% TAVR patients (all *p* < 0.001; Table 1). STS PROM ≤3% SAVR patients were less likely to have had previous coronary artery bypass, percutaneous coronary intervention, and permanent pacemaker (all *p* < 0.001; Table 1).

### 3.2. Clinician Judgment of TAVR Eligibility

Table 2 shows the clinical factors that led heart teams to recommend TAVR in patients with STS PROM ≤3%. More than one factor was given in 44.1% of surveys. Two, three, and four supporting clinical factors were listed in 35.9%, 6.7%, and 1.4% of responses, respectively.

Frailty was the most common clinical factor supporting a TAVR recommendation. Heart team surveys indicated that heart teams defined frailty as either a prolonged 5-meter walk test, use of a cane or walker, and/or wheelchair dependency. Surveys also showed that, among patients recommended for TAVR on the basis of frailty, 14% of these patients were wheelchair-dependent and for those who could walk, the mean 5-meter walk time was 8.7 ± 3.1 seconds. The second most common factor for recommending TAVR was a hostile chest. Among these cases, common conditions included ascending aorta calcification (38% of hostile chest cases), redo sternotomy in an octogenarian (28%), history of chest radiation (14%), and prior left internal mammary artery graft under the sternum (12%). Severe lung disease was the third most common factor for recommending TAVR. Surveys reported that severe pulmonary systolic hypertension (60.1 ± 14.1 mmHg) was present in 35% of patients recommended for this reason and 24% were oxygen-dependent. The fourth most common factor for a TAVR recommendation in patients with STS PROM ≤3% was morbid obesity. Surveys showed that patients recommended for this reason had a mean BMI of 43.7 ± 11.4.

### 3.3. TAVR Procedural Details

Compared to the STS PROM >3% TAVR patients, the STS PROM ≤3% TAVR group was more likely to undergo transfemoral TAVR (93.2% vs. 86.8%; *p* < 0.001) and had shorter procedure time (86.6 ± 43.6 min vs. 94.5 ± 50.4 min, p = 0.001) but used more contrast (115.6 ± 74.0 vs. 103.8 ± 65.4 ml; *p* ≤ 0.001) (Table 3).

### 3.4. Postprocedural Complications and Outcomes

Complications and postprocedural outcomes are shown in Table 4. The in-hospital mortality rate in the STS PROM ≤3% TAVR group was lower at 1.2% that at 2.6% in the STS PROM >3% TAVR group (*p*=0.118) but higher than that in the STS PROM ≤3% SAVR group (1.2% vs. 0.3%; *p*=0.031). Major in-hospital complication rates were similar between the TAVR groups. There was no difference in incidence of a significant cardiac event, requirement for pacer or implantable cardioverter (ICD), stroke, acute kidney injury, disabling or life-threatening bleeding, vascular access site complication, device complication, or moderate to severe aortic regurgitation. In comparison to STS PROM ≤3% SAVR patients, STS PROM ≤3% TAVR patients were less likely to receive a red blood cell transfusion (5.1% vs. 14.3%; *p* < 0.001) but with a higher need for a permanent pacemaker or ICD (6.9% vs. 4.2%; *p*=0.027). Acute kidney injury and stroke rates were similar between these groups.

The STS PROM ≤3% TAVR group had a lower postprocedure hospital stay (2.5 ± 3.4 vs. 3.5 ± 4.7 days; *p* ≤ 0.001) and was more likely to be discharged to home (93.9% vs. 84.6%, *p* < 0.001) than that of the STS PROM >3% TAVR group. In comparison to STS PROM ≤3% SAVR patients, STS PROM ≤3% TAVR patients also had a lower postprocedure hospital stay (2.5 ± 3.4 vs. 5.8 ± 3.4 days; *p* ≤ 0.001) and higher likelihood of discharge to home (93.9% vs 86.8%; *p* < 0.001).

**Figure 3:**
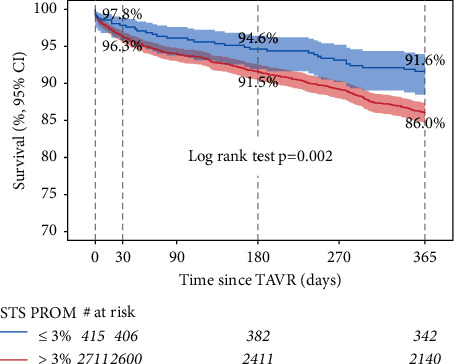
Survival after transcatheter aortic valve replacement surgery in patients with STS PROM ≤3% and STS PROM >3%. Kaplan–Meier survival curves for transcatheter aortic valve replacement (TAVR) surgery patients are shown. Data are extended to one year after TAVR surgery. Patients were grouped according to their Society of Thoracic Surgeons predicted risk of mortality (≤3% or > 3% STS PROM).

The 30-day mortality trended lower in the STS PROM ≤3% TAVR group than in STS PROM >3% TAVR group (2.0% vs. 3.6%; *p*=0.097). In contrast, the 30-day mortality rate was higher for the STS PROM ≤3% TAVR group than the STS PROM ≤3% SAVR group (2.0% vs 0.6%; *p*=0.012). 30-day readmission rates were similar for all groups. In one year (Figure 3), TAVR patients with STS PROM ≤3% had significantly better one-year survival than TAVR patients with STS PROM >3% (91.9% (CI 88.8–94.2%) vs. 86.9% (CI 85.6–88.2%); *p*=0.006).

## 4. Discussion

Results of this study show that heart teams generally overrule STS PROM scores ≤3% for four major reasons: frailty, hostile chest, severe lung disease, and morbid obesity. The cohort of TAVR patients who were recommended in this fashion included significantly more males and younger patients than in the STS PROM >3% group. Incidence of major procedural complications, 30-day mortality, and readmission rates were comparable for both groups, and near-equivalency in these outcomes implies that these cohorts were not essentially different in risk. In fact, the only notable differences in postoperative outcomes were a one-day shorter postprocedural length of stay and higher survival at one year in patients with STS PROM scores ≤3% than in those with an STS PROM >3%. In contrast, TAVR patients with STS PROM ≤3% had higher in-hospital and 30-day mortality rate than the STS PROM ≤3% SAVR patients. Thus, our findings suggest that in cases that were overruled, heart teams choose appropriately and correctly identified high-risk patients. As anticipated, the postprocedure hospital length of stay was higher in the SAVR group associated with a lower likelihood of discharge to home after the procedure.

Evaluating these types of preoperative adjudications is important to understand how and why clinicians exercise this latitude. While the federal eligibility criteria for transcatheter therapies in the United States were evolving, heart teams were permitted to overrule STS PROM scores in cases where the heart team felt that there were extenuating circumstances not accounted by the STS risk model that increased the probability of perioperative mortality. The value of this deference to clinical judgment has not been well studied. No study has carefully measured how and why these overrule decisions are made and how they impacted patient outcomes; however, the SURTAVI study, which compared TAVR to SAVR in patients at intermediate risk, provides some insight [13]. SURTAVI permitted a screening committee to adjudicate cases wherein patients had an STS PROM score ≤3%, but heart teams felt that patients' actual risk was higher. These patients had a higher all-cause mortality or disabling stroke after SAVR than those randomized to TAVR [13], suggesting that they were indeed higher-risk patients. Thus, SURTAVI also supported the importance of clinician judgment as well as the use of risk scores.

Our study provides additional detailed data about the clinical reasons that support such overruling decisions. There were four major clinical conditions that prompted heart teams to believe that patients' risk was higher than STS PROM scores indicated: frailty, hostile chest, lung disease, and obesity. Frailty is a well-recognized predictor of mortality and complications after heart surgery [14–17], and our results showed that heart teams only disagreed with STS PROM scores in severe cases of frailty. On average, cases overruled for this reason had a mean 5-meter walk time of nearly 9 seconds, while 14% were wheelchair bound, indicating advanced disability. Hostile chest and lung disease are now included in a newer TAVR-specific model that estimates risk of 30-day mortality [18], and mediastinal radiation is incorporated into the updated STS PROM model [19,20]. It should be noted that lung disease and obesity, the third and fourth reasons for disagreeing with STS PROM risk scores, respectively, were components of the STS PROM risk model at the time. Thus, these disagreements may highlight a perception among heart teams that these risk factors are not adequately captured by the risk model.

An additional interesting observation of this analysis is that the ability of the local heart to use their discretion to shift low PROM, but suspected high procedural risk, to TAVR appears to have led to overall improved results for low-risk SAVR. In our study, we noted a 30-day mortality of 0.6% compared to the estimated median PROM of 1.3%. Presumably, the improved mortality results reflect diversion of higher risk patients to TAVR. Direct comparison of outcomes between patients with STS PROM ≤3% between TAVR and SAVR is not possible as the TAVR group was older and had a higher incidence of comorbidities as reflected by a higher STS PROM score than that of the SAVR group (2.3% vs 1.3%).

In 2018, an updated STS PROM model was released, which may align more with clinician's judgment for TAVR recommendations [19,20]. In addition, a new TAVR-specific model based on the STS/American College of Cardiology TVT Registry, which incorporates health status and gait speed, has been developed [21]. Both the updated STS PROM model and the STS/American College of Cardiology TAVR model outperformed several other existing models in predicting 30-day post-TAVR mortality [22]. These models may close the gap between clinician judgment and formalized risk scores.

## 5. Conclusion

Our results suggest that heart teams are careful and judicious in overruling risk scores and suggest that as formalized risk models continue to evolve, regulatory provisions should continue to allow heart teams to overrule formalized risk models when deciding between evolving transcatheter therapies and established conventional surgical procedures.

## Figures and Tables

**Table 1:** Baseline demographic and clinical characteristics by study groups. Blank responses in the SAVR group represent data not collected in STS Adult Cardiac Surgery Database.

	TAVR	TAVR	SAVR	*p* value (TAVR, STS PROM > 3% vs. ≤3%)	*p* value (STS PROM ≤ 3%, TAVR vs. SAVR)
	STS PROM ≤ 3% (*N* = 415)	STS PROM > 3% (*N* = 2,711)	STS PROM ≤ 3% (*N* = 1,438)		
STS PROM %, mean ± SD	2.3 ± 0.5	7.2 ± 4.2	1.4 ± 0.7	<0.001	<0.001
STS PROM %, median (IQR)	2.3 (1.9–2.7)	6.0 (4.4–8.6)	1.3 (0.9–1.9)	<0.001	<0.001
Age, mean ± SD	74.4 ± 8.6	82.2 ± 8.4	64.4 ± 11.8	<0.001	<0.001
Male, N (%)	289 (69.6%)	1385 (51.1%)	943 (65.6%)	<0.001	0.123
BSA, median (IQR)	2.0 (1.8–2.1)	1.9 (1.7–2.0)	2.0 (1.9–2.2)	<0.001	0.231
BMI, median (IQR)	28.4 (25.2–34.1)	27.1 (23.7–31.9)	29.1 (25.6–33.2)	<0.001	0.400
Race Caucasian, N (%)	395 (95.2%)	2575 (95.0%)	1305 (92.4%)	0.864	0.052
Permanent pacemaker, N (%)	33 (8.0%)	390 (14.4%)	38 (2.6%)	<0.001	<0.001
Prior ICD, N (%)	6 (1.4%)	93 (3.4%)	11 (0.8%)	0.031	0.200
Prior PCI, N (%)	128 (30.8%)	986 (36.4%)	113 (7.9%)	0.028	<0.001
Prior CABG, N (%)	53 (12.8%)	608 (22.4%)	33 (2.3%)	<0.001	<0.001
Previous AV replacement, N (%)	30 (7.2%)	153 (5.6%)	59 (4.1%)	0.202	0.009
Prior stroke, N (%)	67 (16.1%)	346 (12.8%)	81 (5.6%)	0.059	<0.001
Prior PAD, N (%)	106 (25.5%)	909 (33.5%)	85 (5.9%)	0.001	<0.001
Current/recent smoker, N (%)	39 (9.4%)	163 (6.0%)	142 (9.9%)	0.009	0.761
Hypertension, N (%)	342 (82.4%)	2407 (88.8%)	1039 (72.3%)	<0.001	<0.001
Diabetes, N (%)	118 (28.5%)	1097 (40.5%)	303 (21.1%)	<0.001	0.001
GFR, median (IQR)	77.5 (62.4–97.0)	58.3 (43.5–74.3)	81.3 (68.8–94.1)	<0.001	0.011
Currently on dialysis, N (%)	1 (0.2%)	125 (4.6%)	4 (0.3%)	<0.001	>0.999
Chronic lung disease, moderate/severe, N (%)	52 (12.6%)	599 (22.2%)	30 (2.1%)	<0.001	<0.001
Home oxygen, N (%)	25 (6.0%)	300 (11.1%)	7 (0.5%)	0.002	<0.001
Hostile chest, N (%)	41 (9.9%)	174 (6.4%)		0.009	
Immunocompromise present, N (%)	37 (8.9%)	374 (13.8%)	49 (3.4%)	0.006	<0.001
Prior MI, N (%)	77 (18.6%)	639 (23.6%)	102 (7.1%)	0.023	<0.001
NYHA class III or IV within 2 weeks, N (%)	253 (61.6%)	2006 (74.3%)	233 (16.2%)	<0.001	<0.001
Calcified/atherosclerotic aorta, N (%)	36 (8.7%)	112 (4.1%)	20 (1.4%)	<0.001	<0.001
Left ventricle ejection fraction (%), median (IQR)	61.0 (55.0–65.0)	60.0 (45.0–65.0)	61.0 (58.0–65.0)	<0.001	0.961
Atrial fibrillation/flutter, N (%)	125 (30.1%)	1201 (44.4%)	141 (10.1%)	<0.001	<0.001
Five-meter walk time (sec), median (IQR)	6.7 (5.0–8.0)	7.7 (6.0–9.7)	5.0 (4.0–5.3)	<0.001	<0.001
AV peak velocity m/sec, median (IQR)	4.2 (3.8–4.5)	4.1 (3.6–4.5)		0.002	
AV peak gradient mmHg, median (IQR)	70.0 (58.0–83.0)	67.0 (53.0–80.0)		0.001	
AV annulus diameter size (mm), median (IQR)	24.0 (22.0–26.0)	23.0 (21.0–25.0)		<0.001	
Smallest AV area (cm^2^), median (IQR)	0.8 (0.6–0.9)	0.7 (0.6–0.8)	0.8 (0.7–0.9)	<0.001	0.106
AV mean gradient mmHg, median (IQR)	42.0 (36.0–51.8)	40.0 (31.0–49.0)	45.0 (38.0–55.0)	<0.001	<0.001
AV morphology bicuspid, N (%)	28 (6.8%)	66 (2.4%)		<0.001	
MV regurgitation, moderate/severe, N (%)	56 (13.5%)	672 (24.9%)	114 (8.0%)	<0.001	0.001

Blank responses in the SAVR group represent data not collected in STS Adult Cardiac Surgery Database. Abbreviations: IQR=interquartile range; SD=standard deviation; STS PROM=predicted risk of mortality for surgical aortic valve replacement based on the Society for Thoracic Surgeon's risk model; BSA=body surface area; BMI=body mass index; ICD=implantable cardioverter defibrillator; CABG=coronary artery bypass graft; AV=aortic valve; PAD=peripheral arterial disease; GFR=glomerular filtration rate; MI=myocardial infarction; NYHA=New York Heart Association; MV=mitral valve.

**Table 2:** Reasons for recommending transcatheter aortic valve replacement surgery for patients with STS PROM ≤3%.

Reason	N	% of patients
Total	638	100.0%
Frailty	236	56.9%
Hostile chest	92	22.2%
Severe lung disease	66	15.9%
Obesity	52	12.5%
Risk of stroke	38	9.2%
Cirrhosis	32	7.7%
Bleeding concern	29	7.0%
Malignancy	28	6.7%
Cognitive impairment	22	5.3%
Need for urgent noncardiac surgery	13	3.1%
Malnutrition	9	2.2%
Others	8	1.9%
Unknown	13	3.1%
		
# of factors	N	% of patients
Patients with 1 factor	219	52.8%
Patients with 2 factors	149	35.9%
Patients with 3 factors	28	6.7%
Patients with 4 factors	6	1.4%

More than one reason could be recommended per case. Percent was calculated based on the total number of patients included in this analysis (*n* = 415).

**Table 3:** Procedure information of TAVR groups.

	TAVR	TAVR	*p* value
	STS PROM≤3% (*N* = 415)	STS PROM>3% (*N* = 2,711)	
TAVR access site, N (%)			<0.001
Femoral	389 (94.0%)	2345 (87.0%)	
Transapical	14 (3.4%)	191 (7.1%)	
Transcarotid	6 (1.4%)	73 (2.7%)	
Subclavian/axillary	3 (0.7%)	62 (2.3%)	
Others	3 (0.7%)	40 (1.5%)	
TAVR procedure time (min), mean ± SD	86.6 ± 43.6	94.5 ± 50.4	0.003
TAVR procedure time (min), median (IQR)	74.0 (57.0–104.0)	81.0 (61.0–113.0)	<0.001
Contrast volume (ml), median (IQR)	100.0 (65.0–150.0)	90.0 (60.0–130.0)	0.003
Fluoroscopy time, median (IQR)	13.6 (9.2–20.0)	14.6 (9.9–20.9)	0.115
Type of valve, N (%)			
Single balloon-expandable valve	319 (76.9%)	2023 (74.6%)	0.326
Single self-expanding valve	84 (20.2%)	615 (22.7%)	0.266
Multiple valves	5 (1.2%)	42 (1.5%)	0.591

Abbreviations: SD=standard deviation; IQR=interquartile range; STS PROM=predicted risk of mortality for surgical aortic valve replacement based on the Society for Thoracic Surgeon's risk model; TAVR=transcatheter aortic valve replacement.

**Table 4:** Outcomes by study groups.

	TAVR	TAVR	SAVR	*p* value (TAVR, STS PROM > 3% vs. ≤3%)	*p* value (STS PROM ≤ 3%, TAVR vs. SAVR)
	STS PROM ≤ 3% (*N* = 415)	STS PROM > 3% (*N* = 2,711)	STS PROM ≤ 3% (*N* = 1,438)		

*Postprocedural*					
Transfusion, N (%)	21 (5.1%)	292 (10.8%)	205 (14.3%)	<0.001	<0.001
Units of RBC transfused, mean ± SD	0.2 ± 1.0	0.3 ± 1.2	0.4 ± 1.4	0.029	<0.001
Postoperation length of stay (days), mean ± SD	2.5 ± 3.4	3.5 ± 4.7	5.9 ± 3.6	<0.001	<0.001
Postoperation length of stay (days), median (IQR)	2.0 (1.0–3.0)	2.0 (1.0–4.0)	5.0 (4.0–6.0)	<0.001	<0.001
Discharge to home, N (%)	385 (93.9%)	2235 (84.6%)	1248 (86.8%)	<0.001	<0.001
Death in hospital, N (%)	5 (1.2%)	70 (2.6%)	4 (0.3%)	0.088	0.031
Significant cardiac event, N (%)	5 (1.2%)	46 (1.7%)		0.461	
Stroke, N (%)	5 (1.2%)	57 (2.1%)	11 (0.8%)	0.222	0.394
Acute kidney injury (stage 3), N (%)	3 (0.7%)	52 (2.1%)	13 (0.9%)	0.076	>0.999
Bleeding (disabling or life threatening), N (%)	6 (1.4%)	79 (2.9%)		0.086	
Vascular access site complications, N (%)	17 (4.1%)	160 (5.9%)		0.138	
Device complications, N (%)	4 (1.0%)	26 (1.0%)		>0.999	
Aortic regurgitation (moderate to severe), N (%)	6 (1.5%)	52 (2.1%)	1 (0.7%)	0.463	0.684
Requirement for pacer or ICD, N (%)	26 (6.9%)	216 (9.5%)	58 (4.2%)	0.099	0.027

*30-day follow-up*					
Mortality within 30-day, N (%)	8 (2.0%)	95 (3.6%)	9 (0.6%)	0.097	0.012
Readmission within 30 days, N (%)	36 (8.9%)	269 (10.3%)	107 (7.5%)	0.392	0.349

*1-year follow-up*					
1year survival status, N (%)					
Alive	342 (82.4%)	2138 (78.9%)		0.005	
Died	34 (8.2%)	371 (13.7%)			
Unknown	39 (9.4%)	202 (7.5%)			
Survival at 1 year, % (95% CI)^*∗*^	91.6 (88.5–93.9)	86.0 (84.6–87.2)		0.002	
Readmission free at 1 year, % (95% CI)^	71.5 (66.8–76.0)	71.1 (69.3–72.9)		0.721	

Blank responses in the SAVR group represent data not collected in STS Adult Cardiac Surgery Database. Abbreviations: SD=standard deviation; STS PROM=predicted risk of mortality for surgical aortic valve replacement based on the Society for Thoracic Surgeon's risk model; ICD=implantable cardioverter defibrillator. ^*∗*^Kaplan–Meier estimation; ^cumulative incidence function.

## Data Availability

Data for this project will not be made available because they were gathered from patient records, and data sharing was not requested from or approved by the IRB.

## Abbreviations

AV: Aortic valve
STS: Society of Thoracic Surgeons
PROM: Predicted risk of mortality
TAVR: Transcatheter aortic valve replacement
TVT: Transcatheter valve therapy.

## Supplementary Materials

Supplementary Materials Details on the missing data and the survey data collection instrument that was used in this project are included as supplementary materials.

## References

[1] Kundi H., Strom J. B., Valsdottir L. R. (2018). Trends in isolated surgical aortic valve replacement according to hospital-based transcatheter aortic valve replacement volumes. *JACC: Cardiovascular Interventions*.

[2] Huu A. L., Shum-Tim D. (2020). Transcatheter valve replacement: a revolution. *Future Cardiology*.

[3] Leon M. B., Smith C. R., Mack M. J. (2016). Transcatheter or surgical aortic-valve replacement in intermediate-risk patients. *New England Journal of Medicine*.

[4] Reardon M. J., Van Mieghem N. M., Popma J. J. (2017). Surgical or transcatheter aortic-valve replacement in intermediate-risk patients. *New England Journal of Medicine*.

[5] Popma J. J., Deeb G. M., Yakubov S. J. (2019). Transcatheter aortic-valve replacement with a self-expanding valve in low-risk patients. *New England Journal of Medicine*.

[6] Mack M. J., Leon M. B., Thourani V. H. (2019). Transcatheter aortic-valve replacement with a balloon-expandable valve in low-risk patients. *New England Journal of Medicine*.

[7] FDA News Release (2022). Fda expands indication for several transcatheter heart valves to patients at low risk for death or major complications associated with open-heart surgery. https://www.fda.gov/news-events/press-announcements/fda-expands-indication-several-transcatheter-heart-valves-patients-low-risk-death-or-major.

[8] Carroll J. D., Edwards F. H., Marinac-Dabic D. (2013). The STS-ACC transcatheter valve Therapy national registry. *Journal of the American College of Cardiology*.

[9] The STS (2022). Adult cardiac surgery Database. the society of thoracic Surgeons. https://www.sts.org/registries/sts-national-database/adult-cardiac-surgery-database.

[10] Choudhury J. B. (2002). Non-parametric confidence interval estimation for competing risks analysis: application to contraceptive data. *Statistics in Medicine*.

[11] Gray R. J. (1988). A class of K-sample tests for comparing the cumulative incidence of a competing risk. *Annals of Statistics*.

[12] R Foundation for Statistical Computing (2018). *A Language and Environment for Statistical Computing*.

[13] Serruys P. W., Modolo R., Reardon M. (2018). One-year outcomes of patients with severe aortic stenosis and an STS PROM of less than three percent in the SURTAVI trial. *EuroIntervention*.

[14] Afilalo J., Lauck S., Kim D. H. (2017). Frailty in older adults undergoing aortic valve replacement. *Journal of the American College of Cardiology*.

[15] Thongprayoon C., Cheungpasitporn W., Kashani K. (2017). The impact of frailty on mortality after transcatheter aortic valve replacement. *Annals of Translational Medicine*.

[16] Sepehri A., Beggs T., Hassan A. (2014). The impact of frailty on outcomes after cardiac surgery: a systematic review. *The Journal of Thoracic and Cardiovascular Surgery*.

[17] Revenig L. M., Canter D. J., Taylor M. D. (2013). Too frail for surgery? Initial results of a large multidisciplinary prospective study examining preoperative variables predictive of poor surgical outcomes. *Journal of the American College of Surgeons*.

[18] Arnold S. V., O’Brien S. M., Vemulapalli S. (2018). Inclusion of functional status measures in the risk adjustment of 30-day mortality after transcatheter aortic valve replacement. *JACC: Cardiovascular Interventions*.

[19] Shahian D. M., Jacobs J. P., Badhwar V. (2018). The society of thoracic Surgeons 2018 Adult cardiac surgery risk models: Part 1-background, design considerations, and model development. *The Annals of Thoracic Surgery*.

[20] O’Brien S. M., Feng L., He X. (2018). The society of thoracic Surgeons 2018 Adult cardiac surgery risk models: Part 2-statistical methods and results. *The Annals of Thoracic Surgery*.

[21] O’Brien S. M., Shahian D. M., Filardo G. (2009). The Society of Thoracic Surgeons 2008 cardiac surgery risk models: part 2--isolated valve surgery. *The Annals of Thoracic Surgery*.

[22] Arsalan M., Maren W., Hecker F. (2018). TAVI risk scoring using established versus new scoring systems: role of the new STS/ACC model. *EuroIntervention*.

