# Supplementary material for: Clinical Factors and Outcomes When Real-World Heart Teams Overruled STS Risk Scores in TAVR Cases
Source: J Interv Cardiol. 2022 Jun 25;2022:9926423. doi: 10.1155/2022/9926423 (PMC9252751; doi:10.1155/2022/9926423)
Supplement: Supplementary Materials — Details on the missing data and the survey data collection instrument that was used in this project are included as supplementary materials. [file 9926423.f1.zip › 9926423.f1/ID 9926423 Supplement 1 (Missing Data).docx]

**Supplement**

Handling of missing data

Missing data were not imputed and were excluded from the analysis. Variables that were missing in more than 1% of patient records were as follows. For TAVR patients, 638 patient records were missing time of 5-meter walk (including patients who could not walk), 240 were missing data on post-procedure aortic regurgitation (moderate to severe), 91 were missing Acute Kidney Injury (Stage 3), 131 were missing aortic valve (AV) annulus diameter size, 106 were missing fluoroscopy time, 93 were missing contrast volume, 92 were missing pre-operative AV peak velocity, 90 were missing smallest AV area, 81 were missing AV peak gradient, 57 were missing glomerular filtration rate, 51 were missing pre-operative AV mean gradient. Thirty-day mortality were unknown for 105 patients, 30-day readmission were unknown for 115 patients, 1-year survival status were unknown for 241 patients, and 1-year readmission were unknown for 115 patients. For SAVR patients, 1303 patient records were missing aortic regurgitation (moderate to severe), 1125 were missing time of 5-meter walk (including patients who could not walk), 279 were missing smallest AV area, 266 were missing AV mean gradient, 44 were missing history of atrial fibrillation and/or atrial flutter, 37 were missing chronic lung disease (moderate or severe), 28 were missing 30-day mortality, 26 were missing race, 24 were missing ejection fraction. Thirty-day mortality were unknown for 46 patients, 30-day readmission were unknown for 28 patients.
