# Supplementary material for: Clinical Factors and Outcomes When Real-World Heart Teams Overruled STS Risk Scores in TAVR Cases
Source: J Interv Cardiol. 2022 Jun 25;2022:9926423. doi: 10.1155/2022/9926423 (PMC9252751; doi:10.1155/2022/9926423)
Supplement: Supplementary Materials — Details on the missing data and the survey data collection instrument that was used in this project are included as supplementary materials. [file 9926423.f1.zip › 9926423.f1/ID 9926423 Supplement 2 (Survey Tool).pdf]

Hospital: __________ Patient initials: ______ Patient DOB: ________ Date of TAVR Implant: _________

Please indicate which of the following clinical factors prompted your Heart Team to recommend TAVR despite an STS predicted mortality for isolated AVR of $\leq$ 3.0%. Please list all factors (and available qualifying information).

Please also circle most important factor in this decision.

☐ Frailty: ☐ 6m walk test ___ seconds ☐ Uses walker or cane

☐ Wheelchair dependent ☐ Does not live independently

☐ Malnutrition list albumin level ____

☐ Extreme Body Habitus: ☐ Obesity ☐ Emaciation/small stature list BMI ____

☐ Hostile Chest Anatomy: ☐ Ascending aorta calcifications ☐ Previous chest radiation

☐ Graft or RV under sternum ☐ Previous sternal infection or nonunion

☐ Other list: ____

☐ Severe Pulmonary Disease: list % Predicted FEV1 ____ list % Predicted DLCO ______

☐ Supplemental Oxygen ☐ Nocturnal BiPap

☐ Severe Pulmonary Hypertension: list PA systolic pressure ____ mmHg

☐ Liver Disease: Describe: ____

☐ High Risk of Stroke: Describe: ____

☐ Bleeding Concern: ☐ Thrombocytopenia ☐ Coagulopathy ☐ Jehovah's Witness

Describe: ____

☐ Malignancy: Describe: ____

☐ Need to undergo additional medical procedure in near future:

Describe: ____

☐ Other not listed above:

Describe: ____

____

Please list additional relevant information regarding Heart Team's decision to recommend TAVR for this patient: ____

____
